# Combination of dextromethorphan and memantine in treating bipolar spectrum disorder: a 12-week double-blind randomized clinical trial

**DOI:** 10.1186/s40345-019-0174-8

**Published:** 2020-03-02

**Authors:** Sheng-Yu Lee, Tzu-Yun Wang, Shiou-Lan Chen, Yun-Hsuan Chang, Po-See Chen, San-Yuan Huang, Nian-Sheng Tzeng, Liang-Jen Wang, I-Hui Lee, Kao-Ching Chen, Yen-Kuang Yang, Jau-Shyong Hong, Ru-Band Lu

**Affiliations:** 1 Department of Psychiatry, Kaohsiung Veterans General Hospital, Kaohsiung, Taiwan; 2 Department of Psychiatry, School of Medicine, College of Medicine, Kaohsiung Medical University, Kaohsiung, Taiwan; 3 Department of Psychiatry, College of Medicine, National Yang-Ming University, Taipei, Taiwan; 4 Department of Psychiatry, National Cheng Kung University Hospital, College of Medicine, National Cheng Kung University, 138 Sheng-Li Road, Tainan, 70428 Taiwan; 5 Department of Neurology, School of Medicine, Kaohsiung Medical University, Kaohsiung, Taiwan; 6 Department of Psychology, College of Medical and Health Science, Asia University, Taichung, Taiwan; 7 Department of Psychiatry, Tri-Service General Hospital, National Defense Medical Center, Taipei, Taiwan; 8 Department of Child and Adolescent Psychiatry, Kaohsiung Chang Gung Memorial Hospital and Chang Gung University College of Medicine, Kaohsiung, Taiwan; 9 Laboratory of Toxicology and Pharmacology, NIH/NIEHS, Research Triangle Park, Durham, NC USA; 10 Yanjiao Furen Hospital, Hebei, China; 11 Addiction Research Center, National Cheng Kung University, Tainan, Taiwan

**Keywords:** Bipolar spectrum disorder, Bipolar II disorder, Dextromethorphan, Memantine, Cytokines, BDNF

## Abstract

**Background:**

The aim of this study is to determine whether adding combination of agents with anti-inflammatory and neurotrophic effects is more efficacious than mood stabilizer alone in improving clinical symptoms, plasma brain-derived neurotrophic factor (BDNF), cytokine levels, and metabolic profiles in patients with bipolar spectrum disorder.

**Methods:**

In a randomized, double-blind, controlled 12-week clinical trial, patients with moderate mood symptoms (HDRS ≥ 18 or YMRS ≥ 14) were recruited. The patients were randomly assigned to a group while still undergoing regular valproate (VPA) treatments: VPA + dextromethorphan (DM) (30 mg/day) + memantine (MM) (5 mg/day) (DM30 + MM5) (n = 66), VPA + DM (30 mg/day) (DM30) (n = 69), VPA + MM (5 mg/day) (MM5) (n = 66), or VPA + Placebo (Placebo) (n = 69). Symptom severity, immunological parameters [plasma tumor necrosis factor (TNF)-α and C-reactive protein (CRP)] and plasma brain-derived neurotrophic factor (BDNF) were regularly examined. Metabolic profiles [cholesterol, triglycerides, glycosylated hemoglobin (HbA1C), fasting serum glucose, body mass index (BMI)] were measured at baseline and at 2, 8, and 12 weeks.

**Results:**

Depression scores were significantly (*P* = 0.03) decreases and BDNF levels significantly (*P* = 0.04) increased in the DM30 + MM5 group than in the Placebo group. However, neither depressive scores nor BDNF levels were significantly different between the DM30, MM5, and Placebo groups. Changes in certain plasma cytokine and BDNF levels were significantly correlated with metabolic parameters.

**Conclusion:**

We concluded that add-on DM30 + MM5 was significantly more effective than placebo for clinical symptoms and plasma BDNF levels. Additional studies with larger samples and mechanistic studies are necessary to confirm our findings.

*Trial registration* NCT03039842 (https://register.clinicaltrials.gov/). Trial date was from 1 Jan 2013 to 31 December 2016 in National Cheng Kung University Hospital. Registered 28 February 1 2017-Retrospectively registered, https://clinicaltrials.gov/ct2/show/NCT03039842?term=NCT03039842&rank=1.

## Background

Bipolar II disorder (BD-II) is a subtype of bipolar disorder (BD) characterized as recurrent episodes of depression and hypomania. BD-II is frequently misdiagnosed in clinical settings because the diagnosis is frequently made based on recollections of past hypomania while patients present with symptoms of depression (Mosolov et al. 2014). A consequence of the frequent misdiagnosis or delayed diagnosis of BD-II is ineffective treatment (Drancourt et al. 2013): Patients with BD-II are at a greater risk of suicide, a prolonged clinical course, more mood episodes, more major and minor depressive episodes, and shorter interepisode intervals, than are patients with BD-I (Karanti et al. 2019).

Neurodegeneration and inflammation are considered the pathogenesis of BD (Berk et al. 2011; Drevets 2000; Peng et al. 2005). Brain-derived neurotrophic factor (BDNF) is important for the development and survival of monoamine neurons (Cotman and Berchtold 2002). Several lines of evidence suggest the involvement of BDNF in the pathogenesis of mood disorders and the mechanism of action of mood stabilizing medication, which indicates probable neurotrophic and neuronal dysfunction in patients with BD (Duman 2002; Mora et al. 2019). Two meta-analyses reported significantly lower-than-normal-range serum BDNF levels in patients with BD during episodes of mania and depression, but not different from controls during euthymic state (Fernandes et al. 2015; Munkholm et al. 2016). Level of BDNF increased after patients were treated for mood episode (Tunca et al. 2014). Plasma BDNF levels might also be associated with the degree of neurodegeneration in BD (de Oliveira et al. 2009). Because BDNF can cross the blood–brain barrier (Karege et al. 2005), BDNF levels in the brain and plasma might undergo similar changes during maturation and aging in vivo (Cirulli et al. 2009). Therefore, plasma BDNF levels might reflect BDNF levels in the human brain (Pan et al. 1998; Klein et al. 2011). In addition, peripheral BDNF has been proposed as a candidate biomarker of mood states and disease progression for BD (Fernandes et al. 2011).

Increasing evidence suggests that abnormal cytokine-mediated inflammatory responses might be another pathogenesis of BD (Goldstein et al. 2009; Rosenblat et al. 2016). Strakowski et al. (2000) proposed that subcortical structural abnormalities in the striatum, amygdala, and prefrontal cortex might lead to neuroinflammation and then contribute to the progression of brain atrophy and exacerbation of the symptoms of BD. Meta-analyses have also reported higher-than-normal levels of the proinflammatory cytokine tumor necrosis factor (TNF)-α and of C-reactive protein (CRP) during acute manic and depressive episodes; both substances decreased after treatment (Fernandes et al. 2016; Goldsmith et al. 2016). Cytokines have also been proposed as diagnostic and staging biomarkers for BD (Goldstein et al. 2015; Jacoby et al. 2016).

Patients with BD appear to have a higher prevalence rate of metabolic syndrome than does the general population (Vancampfort et al. 2013). In BD, obesity has been associated with greater illness severity and cognitive dysfunction, and even suicide attempts (Goldstein et al. 2011; Yim et al. 2012). One main link between metabolic disturbance and BD was postulated as inflammation (Yamagata et al. 2017). Manipulating central and peripheral BDNF levels modified metabolic dysfunction in an animal study (Lyons et al. 1999). BDNF was also suggested to play a crucial role in the pathophysiology of obesity and metabolic syndrome in neurodegenerative disorder such as depression (Motamedi et al. 2017) because low plasma levels of BDNF were reported in patients with metabolic syndrome (Chaldakov et al. 2004). Therefore, the relationship between metabolic abnormality and BD might be a consequence of aberrant inflammatory and neural networks. It is of interest to investigate the correlation between cytokines and BDNF with the metabolic profile in BD.

Although the treatment guidelines for BD are well established (Yatham et al. 2018), many patients remain symptomatic while on mood stabilizers (Rybakowski 2013). It is possible that current mood stabilizers might not prevent ongoing inflammation and neurodegeneration. Therefore, adding agents with anti-inflammatory and neurotrophic effects to current therapy for BD might provide more effective therapy. Both low-dose DM and MM have anti-inflammatory and neurotrophic effects.

DM is an antitussive drug used for more than 50 years (Turchan-Cholewo et al. 2009). DM is a *N*-methyl-d-aspartate (NMDA) receptor antagonist which was reported to be useful at a high dose (480 mg/day) to reduce methadone tolerance in opioid-dependent patients (Cornish et al. 2002). DM also protects monoaminergic neurons against inflammation-mediated degeneration (Liu and Hong 2003) and endotoxicity (Liu and Hong 2003; Zhang et al. 2004; Zhang et al. 2005). Apart from its being an NMDA receptor, DM and its metabolite may stimulate sigma-1 receptor, inhibit serotonin reuptake, and block L-type calcium channel mediating anti-inflammation, anti-convulsive, anti-parkinsonian, anti-ischemic, antioxidant, and alleviation of pseudobulbar effects (Chen 2011; Shin et al. 2011). Above evidence suggests that DM offers promising neuroprotective and anti-inflammatory benefits in neurodegenerative disorders such as are BD. Memantine is a noncompetitive NMDA receptor antagonist. It is also an alternative mechanism: anti-inflammatory effect that downregulates the activity of microglia and upregulates astroglia-released of neurotrophic factors (Wu et al. 2009).

In one of our prior randomized clinical trials, we added low-dose DM (30 mg or 60 mg) or placebo to the pharmacological therapy of BD patients for 12 weeks (Chen et al. 2014); in another, we added low-dose MM (5 mg/day) or placebo (Lee et al. 2013). Although there was no significant improvement in clinical symptoms, the add-on agents reduced the levels of inflammatory markers and increased the levels of plasma BDNF. We then wondered whether the combination effect of DM and MM, both which provide anti-inflammatory and neurotrophic effects, might benefit patients with BD-II more than adding only one of them.

Therefore, we conducted a double-blind, placebo-controlled study of add-on low-dose DM (30 mg) (DM30), MM (5 mg/day) (MM5), DM30 + MM5, or placebo to evaluate their efficacy as augmenting agents in treating BD-II. The outcome measures included clinical severity, levels of cytokine and plasma BDNF expression, and metabolic profiles. In addition, we investigated the correlation between changes in plasma cytokines and BDNF with mood symptoms and metabolic indices over the 12-week pharmacological intervention.

## Methods

The Institutional Review Board for the Protection of Human Subjects at National Cheng Kung University Hospital reviewed and approved the research protocol. All participants were given a detailed description of the study before they signed written informed consent forms.

Patients with BP-II were recruited from outpatient and inpatient settings. All patients were initially evaluated by an attending psychiatrist, once diagnosed with BP-II, they then received a structural interview conducted by a clinical psychologist to confirm the Diagnostic and Statistical Manual of Mental Disorders, fourth edition (DSM-IV) diagnoses, using the Chinese Version of the Modified Schedule of Affective Disorder and Schizophrenia-Life Time (SADS-L) (Endicott and Spitzer 1978), which has good inter-rater reliability (Huang et al. 2004). Patients with other mood and psychotic disorders, borderline personality disorder, drug dependence, or cognitive disorders other than BD-II were excluded. Patients who had taken DM or MM within 1 week before the first dose of the double-blind medication were also excluded. In addition, due to safety reasons, women of childbearing potential not using adequate contraception, pregnant or nursing females, and those who have clinically significant medical condition (i.e. cardiac, hepatic and renal disease) were excluded.

Although DSM-IV-TR criteria require a minimum of 4 days of hypomania, current epidemiologic data suggest that 2 days is more prevalent in community samples; therefore, we used the 2-day minimum for hypomania in the diagnosis of BD-II. However, because some patients may not fulfill criteria of BD-II according to the DSM-IV-TR criteria, it is better to refer our study group as “bipolar spectrum patients” to avoid misleading.

### Study design

After they had been initially screened, the patients were randomly assigned to add-on DM30 group (30 mg/day), the add-on MM5 group (5 mg/day), the add-on DM30 + MM5 group, or to the add-on Placebo group for 12 weeks, during which they were also given open-label VPA treatment [500 mg and 1000 mg daily (50–100 μg/ml in plasma)], which had begun when they joined the study. All patients were prescribed with VPA treatment when they agreed to join the study. It will than take about 1 week for the patients to pass initial screen and undergo randomization for add-on experimental drugs. Therefore, the patients usually receive VPA about 1 week before they were given the experimental drugs. We hired a third party local pharmacological company to prepare the trial medication and perform randomization. They had constructed a combined capsule of either DM30, MM5 or DM30 + MM5 and placebo. The randomization method was block randomization. The add-on medication was delivered by research assistant to the patients after doctor visit. Both the clinician and the research assistants were blind to the add-on therapy throughout the trial. Clinical psychologists with good inter-rater reliability managed the rating of symptoms during each visits in the follow-up duration. Symptom severity was assessed at baseline, and treatment responses were measured on day 7 of weeks 1, 2, 4, 8, and 12. The severity of mood symptoms was assessed using the Hamilton Depression Rating Scale (HDRS) and the Young Mania Rating Scale (YMRS). Only patients with moderate mood symptoms (HDRS ≥ 18 or YMRS ≥ 14) were recruited. Concomitant benzodiazepine medication (lorazepam < 8 mg) was used for nighttime sedation, and to treat agitation and insomnia during the study. Up to 20 mg/daily of fluoxetine was permitted for associated depressive symptoms. Risperidone (1–6 mg/day) were allowed for agitation.

Ten milliliters of whole blood was withdrawn from the antecubital vein of each patient when clinical severity was assessed, at baseline, and on day 7 of weeks 1, 2, 4, 8, and 12. Plasma, which was isolated from the whole blood after it had been centrifuged at 3000*g* for 15 min at 4 °C, was immediately stored at − 80 °C. Cytokine levels were quantified using an antibody pair assay system (Flexia; BioSource Intl., Camarillo, CA). Sample processing and data analysis were done according to the manufacturer’s instructions. The immunological parameters plasma TNF-α and CRP and plasma BDNF were analyzed.

### Statistical analysis

The demographic and clinical characteristics of the patients, their baseline YMRS and HDRS scores, and their baseline cytokine levels were compared between groups using one-way analysis of variance (ANOVA) for continuous variables, and χ^2^ tests for categorical variables. Data are mean ± standard deviation (SD). Both CRP and TNF-α levels were distributed erratically and showed a significant level of positive skew (Table 1). Arithmetic transformations were used to produce approximately normal distributions for further analysis; log (*x *+ 1) was used for cytokine levels.

**Table 1:** Characteristics of the BD-II patients in different treatment groups at baseline

Treatment groups	DM30	MM5	DM30 + MM5	Placebo	F or χ^2^	*P*-value
	(n = 69)	(n = 66)	(n = 66)	(n = 69)		
Age (years)	36.3 ± 13.3	37.4 ± 14.0	35.7 ± 13.2	35.2 ± 13.8	0.32	0.81
Gender (M/F)	34/35	27/39	22/44	34/35	4.83	0.19
Age at onset (years)	15.4 ± 5.0	14.8 ± 5.0	14.9 ± 4.3	13.6 ± 2.8	1.79	0.15
HDRS	15.3 ± 4.2	15.8 ± 4.2	15.9 ± 3.8	15.2 ± 3.7	0.51	0.68
YMRS	13.1 ± 3.2	13.5 ± 2.9	13.5 ± 3.4	13.6 ± 2.8	0.34	0.80
TNF-α (pg/ml)	1.7 ± 1.3	2.1 ± 1.8	1.8 ± 1.3	1.9 ± 1.6	0.88	0.45
CRP (ng/ml)	1658.4 ± 1346.6	1635.4 ± 1237.1	1814.5 ± 1489.6	1878.0.5 ± 1525.0	0.47	0.70
BDNF (ng/ml)	12.6 ± 7.3	12.6 ± 6.9	12.1 ± 6.3	14.0 ± 6.6	0.90	0.44
TC (mg/dl)	179.8 ± 44.9	172.5 ± 29.9	182.0 ± 39.9	180.9 ± 39.8	0.71	0.50
HDL-C (mg/dl)	55.9 ± 15.2	57.5 ± 16.6	59.4 ± 12.7	55.0 ± 17.9	1.00	0.40
LDL-C (mg/dl)	117.4 ± 33.4	109.0 ± 28.0	117.5 ± 36.5	114.2 ± 31.4	0.97	0.41
Triglycerides (mg/dl)	106.0 ± 68.5	94.6 ± 51.2	91.8 ± 44.1	119.6 ± 86.4	1.84	0.14
FBS (mg/dl)	96.1 ± 37.7	88.8 ± 9.6	88.2 ± 13.7	92.7 ± 22.5	1.60	0.19

All data are mean ± SD unless otherwise stipulated

*SD* standard deviation, *HDRS* 17-item Hamilton Depression Rating Scale, *YMRS* Young Mania Rating Scale, *TNF-α* tumor necrosis factor α, *CRP* C-reactive protein, *BDNF* brain-derived neurotrophic factor, *TC* total cholesterol, *HDL-C* high-density lipoprotein-cholesterol, *LDL-C* low-density lipoprotein-cholesterol, *DM30* dextromethorphan (30 mg/day), *MM5* memantine (5 mg/day), *DM30 + MM5* dextromethorphan (30 mg/day) combined with memantine (5 mg/day), *FBS* fasting blood sugar

The intent to-treat (ITT) analysis set included all patients who had taken at least one dose of a study drug, and had undergone one baseline assessment and at least one post-baseline assessment. All outcome variables—HDRS, YMRS, cytokine levels, plasma BDNF levels, and metabolic parameters—in the ITT set were analyzed. Missing data were filled in using the last observation carried forward (LOCF) method.

The overall within-group changes of each outcome over the 12 weeks were analyzed using paired-sample *t*-tests. To compare the difference in outcomes of add-on medication (DM30, MM5, and DM30 + MM5) versus placebo, the generalized estimating equation (GEE) was used. We ran 10 models with each outcome as a dependent variable. In each model, add-on medication (DM30, MM5, and DM30 + MM5 vs. Placebo), treatment duration, treatment given × treatment duration, gender, and age were included as independent variables. The covariance structure used was the autoregressive (AR (Mosolov et al. 2014)) model. The placebo group was the reference group. The interaction term of treatment given × treatment duration was regarded as the effect of add-on DM30, MM5, and DM30 + MM5. To analyze correlations between cytokines, BDNF, and metabolic profiles, GEE analysis was also used. Plasma cytokines and BDNF were set as independent variables and metabolic profile components were set as dependent variables; The analyses were controlled for time effects (treatment period from baseline to week 12), gender and age, and clinical severity. By setting clinical outcomes (HDRS and YMRS) to remission (HDRS ≤ 7 and YMRS < 8), response (change of HAMD or YMRS ≥ 50%) and non-response/remission (Tohen et al. 2009), survival analysis was conducted according to the above defined categorical data and time to response or to remission. The response and remission rate was estimated using the Kaplan–Meier product-limit estimate method, and survival curves were compared using the Cox regression. SPSS 22.0 for Windows was used for all statistical computations. Significance was set at P < 0.05.

## Results

**Fig. 1:**
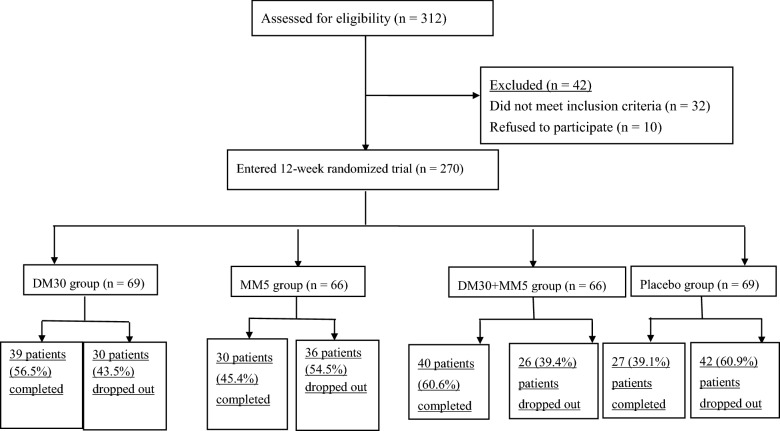
CONSORT diagram

The timeframe for inclusion was from 1 Jan 2013 to 31 December 2016. We initially screened 312 patients: 32 were excluded because they did not meet the inclusion criteria and 10 because they refused to participate. We then randomly assigned 270 BD-II patients to the DM30 (n = 69), MM5 (n = 66), DM30 + MM5 (n = 66), and Placebo (n = 69) groups for 12 weeks. One hundred thirty-six (50.4%) of the 270 patients completed the double-blind phase, and 134 (49.6%) dropped out (Fig. 1): MM5 group: dizziness = 1, refusal of follow-up = 35; DM30: refusal of follow-up = 30; DM30 + MM5: refusal of follow-up = 26; Placebo: refusal of follow-up = 42. Throughout the trial, only one patient in the MM5 group reported dizziness and dropped out from the trial on week 2.

Patient demographic and clinical characteristics, HDRS and YMRS scores, cytokine and BDNF levels, and metabolic profiles of the patients were similar at baseline in the 4 treatment groups at baseline (Table 1). After 12 weeks of treatment, mean within-group changes in clinical severity (HDRS and YMRS scores) were significant in all treatment groups (Table 2). CRP levels were significantly lower in the DM30 + MM5 group, and BDNF levels were significantly higher in the DM30 and DM30 + MM5 groups. Total cholesterol and low density lipoprotein-cholesterol (LDL-C) levels were significantly lower in the Placebo group.

**Table 2:** Within-group changes (baseline minus endpoint) in clinical characteristics of the BD-II patients in different treatment groups after 12 weeks of follow-up

Treatment groups	DM30		MM5		DM30 + MM5		Placebo	
Change	Mean ± SD	*P*-value	Mean ± SD	*P*-value	Mean ± SD	*P*-value	Mean ± SD	*P*-value
Primary outcomes								
HDRS	6.4 ± 4.7	< 0.001**	6.3 ± 4.9	< 0.001**	6.6 ± 4.6	< 0.001**	5.0 ± 4.1	< 0.001**
YMRS	4.7 ± 3.4	< 0.001**	4.6 ± 3.9	< 0.001**	4.9 ± 4.0	< 0.001**	3.6 ± 4.1	< 0.001**
Cytokines and BDNF								
TNF-α (pg/ml)	0.1 ± 2.5	0.76	0.3 ± 1.8	0.09	0.3 ± 1.4	0.16	– 0.2 ± 1.6	0.87
CRP (ng/ml)	10.9 ± 1290.3	0.20	– 12.6 ± 1269.4	0.98	371.3 ± 1157.9	0.005**	20.9 ± 978.8	0.17
BDNF (ng/ml)	– 2.3 ± 8.3	0.035*	0.3 ± 7.6	0.75	– 2.2 ± 8.2	0.044*	0.09 ± 8.9	0.94
Metabolic parameters								
TC (mg/dl)	− 0.1 ± 28.7	0.98	0.5 ± 24.5	0.90	0.6 ± 28.6	0.89	8.3 ± 24.6	0.034*
HDL-C (mg/dl)	1.6 ± 10.2	0.28	2.4 ± 8.7	0.10	− 10.7 ± 82.4	0.41	1.3 ± 7.2	0.26
LDL-C (mg/dl)	− 0.9 ± 23.1	0.79	2.6 ± 22.0	0.48	4.6 ± 24.4	0.23	7.7 ± 21.9	0.034*
Triglycerides (mg/dl)	− 11.6 ± 168.4	0.68	− 10.7 ± 38.7	0.17	− 2.5 ± 44.3	0.73	− 14.0 ± 57.0	0.20
FBS (mg/dl)	9.0 ± 38.1	0.10	− 3.3 ± 22.6	0.33	2.8 ± 9.9	0.06	2.0 ± 14.2	0.34

The difference between baseline and endpoint after 12 weeks of follow-up analyzed using a paired sample *t*-test

*SD* standard deviation, *HDRS* 17-item Hamilton Depression Rating Scale, *YMRS* Young Mania Rating Scale, *TNF-α* tumor necrosis factor α, *CRP* C-reactive protein, *BDNF* brain-derived neurotrophic factor, *TC* total cholesterol, *HDL-C* high-density lipoprotein-cholesterol, *LDL-C* low-density lipoprotein-cholesterol, *DM30* dextromethorphan (30 mg/day), *MM5* memantine (5 mg/day), *DM30 + MM5* dextromethorphan (30 mg/day) combined with memantine (5 mg/day), *FBS* fasting blood sugar

*P < 0.05; **P < 0.01

**Fig. 2:**
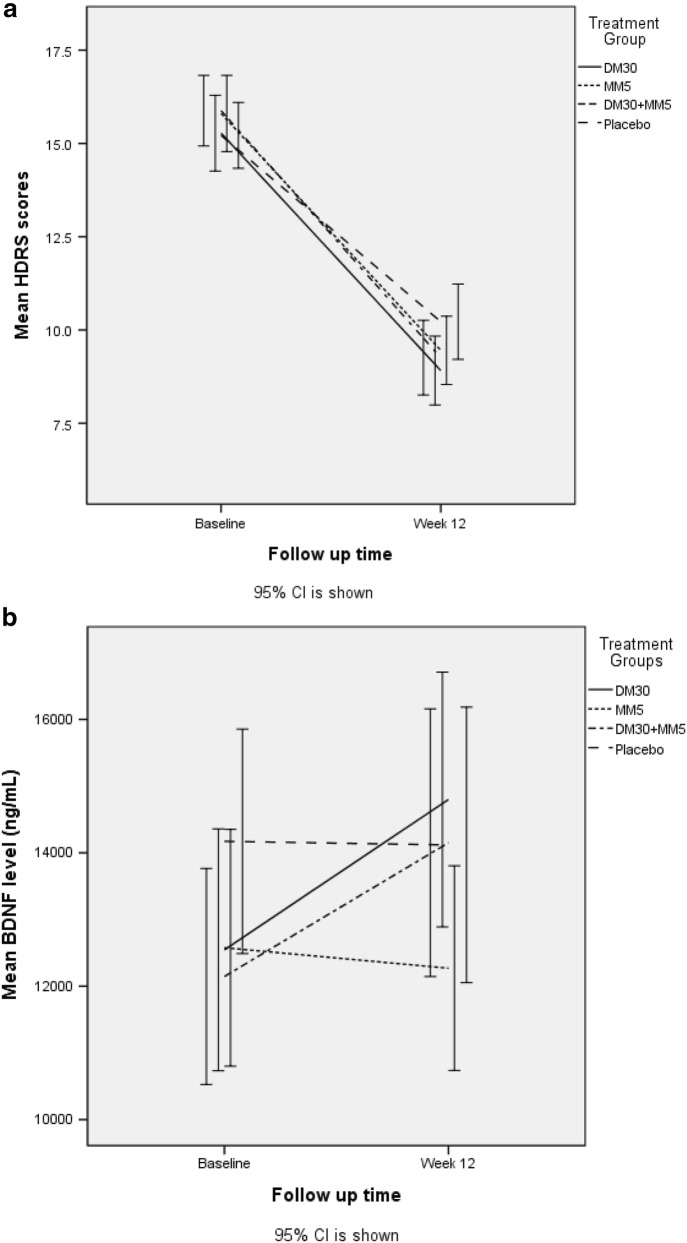
**a** Difference in symptoms of depression in BD-II patients taking add-on DM30, MM5, and DM30 + MM5 versus placebo. **b** Difference in plasma BDNF levels in BD-II patients taking add-on DM30, MM5, and DM30 + MM5 versus placebo

GEE analysis showed a significant change in the HDRS score in the DM30 + MM5 group compared with that in the Placebo group (*P* = 0.03) (Table 3; Fig. 2a); a trend of difference in the change in the YMRS score in the DM30 + MM5 group compared with that in the Placebo group was also noted, though not significant. BDNF levels were significantly higher in the DM30 + MM5 group than in the Placebo group (*P* = 0.04) (Fig. 2b).

**Table 3:** Difference in outcomes of add-on medication (DM30, MM5, and DM30 + MM5) versus Placebo in BD-II patients

	B	Wald χ^2^	*P*-value
HDRS^a^			
DM30	− 1.38	3.36	0.07
MM5	− 1.33	2.96	0.09
DM30 + MM5	− 1.60	4.61	0.03*
YMRS^a^			
DM30	− 1.00	2.44	0.12
MM5	− 0.98	2.07	0.15
DM30 + MM5	− 1.32	3.68	0.06
TNF-α^b^			
DM30	− 0.04	0.14	0.71
MM5	− 0.15	2.00	0.16
DM30 + MM5	− 0.07	0.68	0.41
CRP^b^			
DM30	0.05	0.40	0.53
MM5	0.09	1.18	0.28
DM30 + MM5	− 0.05	0.41	0.52
BDNF^b^			
DM30	2.94	2.97	0.09
MM5	0.02	< 0.001	0.99
DM30 + MM5	3.44	4.23	0.04*
TC^b^			
DM30	6.95	1.60	0.21
MM5	3.14	0.38	0.54
DM30 + MM5	4.37	0.60	0.44
HDL-C^b^			
DM30	0.79	0.11	0.74
MM5	− 0.07	0.001	0.98
DM30 + MM5	14.45	1.04	0.31
LDL-C^b^			
DM30	8.80	3.35	0.07
MM5	4.45	0.80	0.37
DM30 + MM5	3.21	0.38	0.54
Triglycerides^b^			
DM30	28.08	0.94	0.33
MM5	8.81	0.29	0.59
DM30 + MM5	11.69	0.52	0.47
FBS^b^			
DM30	− 5.33	0.90	0.34
MM5	7.63	2.82	0.09
DM30 + MM5	− 0.31	0.02	0.90

*B* standardized coefficient, *HDRS* 17-item Hamilton Depression Rating Scale, *YMRS* Young Mania Rating Scale, *TNF*-*α* tumor necrosis factor α, *CRP* C-reactive protein, *BDNF* brain-derived neurotrophic factor, *TC* total cholesterol, *HDL*-*C* high-density lipoprotein-cholesterol, *LDL*-*C* low-density lipoprotein-cholesterol, *DM30* dextromethorphan (30 mg/day), *MM5* memantine (5 mg/day), *DM30 *+ *MM5* dextromethorphan (30 mg/day) combined with memantine (5 mg/day), *FBS* fasting blood sugar

*P < 0.05

^a^Using the Placebo group as a reference group, controlling for age, gender, and treatment duration. The data shown are the interaction term, treatment group, and treatment duration

^b^Using the Placebo group as a reference group, controlling for age, gender, treatment duration, HDRS, and YMRS. The data shown are the interaction term, treatment group, and treatment duration

**Fig. 3:**
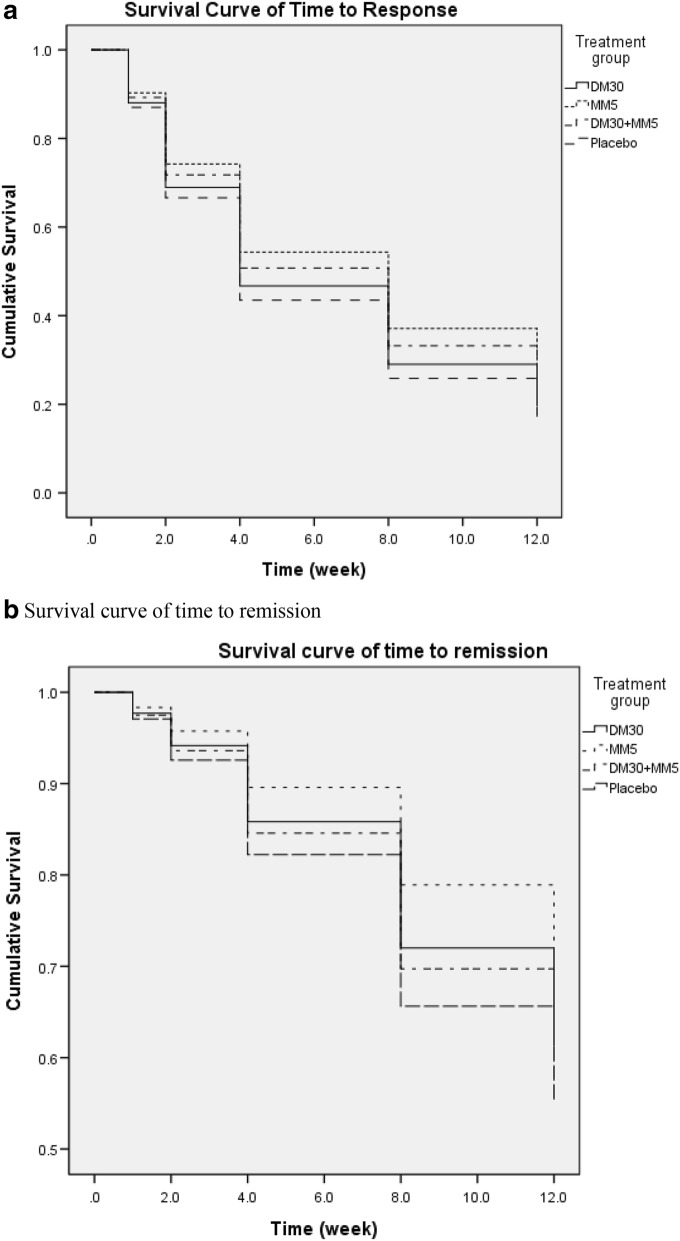
**a** Survival curve of time to response in BD-II patients taking add-on DM30, MM5, and DM30 + MM5 versus placebo. **b** Survival curve of time to response in BD-II patients taking add-on DM30, MM5, and DM30 + MM5 versus placebo

The change in plasma BDNF level was significantly correlated with the changes in total cholesterol (*P* = 0.04) levels. The change in CRP level was significantly correlated with the change in YMRS scores (*P* = 0.03) and high-density lipoprotein-cholesterol (HDL-C) (*P* = < 0.001) and triglyceride levels (*P* ≤ 0.001). The change in TNF-α level was also significantly associated with change in HDL-C (*P* = 0.01) and triglyceride levels (*P* = 0.001) (Table 4). As categorical analysis, we found no significance in the four treatment groups in either response nor remission rate (Table 5, Fig. 3). The frequency of concomitant use of medication are: fluoxetinen (7.8%), lorazepam (89.4%) and risperdone (80.8%). We further analyzed the difference in addition of fluoxetine, benzodiazepine, or risperdone in responders and non-responders. The addition of fluoxetine (P = 0.95), benzodiazepines (P = 0.53) or risperidone (P = 0.77) was not significantly different in those who improved and those who did not improve.

**Table 4:** Correlation between TNF-α, CRP, and BDNF with clinical symptoms and metabolic parameters

	TNF-α			CRP			BDNF		
	B	Wald χ^2^	*P*-value	B	Wald χ^2^	*P*-value	B	Wald χ^2^	*P*-value
HDRS	0.45	1.07	0.30	0.22	0.62	0.43	0.11	1.94	0.16
YMRS	0.20	0.32	0.57	0.72	4.82	0.03*	− 0.01	0.30	0.58
TC	− 1.83	0.28	0.60	1.98	0.32	0.58	0.52	4.20	0.04*
HDL-C	− 5.34	6.24	0.01*	− 11.61	24.82	< 0.001**	0.02	0.02	0.89
LDL-C	− 1.91	0.41	0.53	5.51	3.19	0.07	0.38	2.69	0.10
Triglycerides	22.78	10.67	0.001**	34.58	22.38	< 0.001**	0.97	3.46	0.06
FBS	− 6.37	1.30	0.26	0.73	0.13	0.72	0.27	3.41	0.07

*TNF*-*α* tumor necrosis factor α, *CRP* C-reactive protein, *BDNF* brain-derived neurotrophic factor, *B* standardized coefficient, *HDRS* 17-item Hamilton Depression Rating Scale, *YMRS* Young Mania Rating Scale, *TC* total cholesterol, *HDL*-*C* high-density lipoprotein-cholesterol, *LDL*-*C* low-density lipoprotein-cholesterol, *FBS* fasting blood sugar

*P < 0.05; **P < 0.01

**Table 5:** Survival analysis according to response and remission rate

	Response rate N/total N (%)	Hazard Ratio	95.0% CI	P	Remission rate N/total N (%)	Hazard Ratio	95.0% CI	P
DM30	44/69 (63.7%)	0.92	0.59–1.43	0.70	18/69 (26.1%)	0.78	0.40–1.52	0.46
MM5	34/66 (51.5%)	0.73	0.46–1.18	0.20	12/66 (18.2%)	0.56	0.27–1.18	0.13
DM30 + MM5	39/66 (59.1%)	0.82	0.51–1.29	0.39	19/66 (28.8%)	0.86	0.44–1.65	0.64
Placebo	39/69 (56.5%)	1 (ref)			17/69 (24.6%)	1 (ref)

The data that support the findings of this study are available from the corresponding author upon reasonable request.

## Discussion

Our most important finding was that add-on DM30 + MM5 was significantly more effective than was placebo for improving HDRS scores (depressive symptoms) and raising plasma BDNF levels. However, the treatment effects of add-on DM30 only and of add-on MM5 only were not significantly different from those of placebo. We also found a significant correlation between changes in cytokine and BDNF levels and in metabolic profiles. However, changes in plasma BDNF levels and HDRS scores were not correlated. Our study provides the first evidence that combination of low-dose DM and MM attenuated symptoms of depression in patients with BD-II.

The treatment effects of DM and MM alone on depressive symptoms remains inconsistent. A recent meta-analysis (Kishi et al. 2017) reported that MM alone had no therapeutic effect on major depressive disorder or BD. We previously reported (Wu et al. 2009; Lee et al. 2014) that low-dose MM (5 mg), though had no significant effect on depressive symptoms in BD-II, showed anti-inflammatory and neurotrophic effects. Lauterbach (2016), however, reported that high-dose (60 mg bid) DM had an antidepressant effect in treatment-refractory depression, and Kelly and Lieberman (2014) said that DM (20 mg) + quinidine (10 mg) once or twice a day attenuated BD-II depression. Others have reported that DM protected monoaminergic neurons against inflammation-mediated degeneration (Liu and Hong 2003) and endotoxicity (Liu and Hong 2003; Zhang et al. 2004; Zhang et al. 2005). We reported (Chen et al. 2014) that low-dose (60 mg/day) add-on DM was not more effective than placebo in treating BD, however, we found that DM significantly raised plasma BDNF levels in BD patients after 12 weeks of treatment. No previous studies reported the therapeutic effect of DM + MM in depressive symptoms. From our past study, we propose that the possible mechanism of the therapeutic efficacy of add-on low-dose DM + MM may be related to anti-inflammation and neurotrophic effects. However, since we found no correlation between HDRS and BDNF nor other cytokines, this hypothesis requires additional investigations.

We found no significant changes in metabolic parameters between the add-on treatment groups and the Placebo group. Such changes are affected by various factors—diet, exercise, daily activities, smoking, and underlying body composition—all of which were difficult to control for in the current study because most of our participants were outpatients. It would be ideal if we could control their daily caloric intake and consumption in future studies. Furthermore, we did not find significantly lower TNF-α or CRP levels in the treatment groups than in the Placebo group. We previously reported (Lee et al. 2014), in an independent sample of 214 participants, that MM (5 mg) alone might significantly decrease plasma TNF-α levels compared with placebo. Our current negative finding might be attributable to a smaller sample.

We also found a significant association between changes in plasma BDNF levels and changes in total cholesterol (TC) levels; changes in CRP levels and changes in YMRS scores; changes in CRP levels and changes in HDL-C and triglyceride levels; and changes in TNF-α levels and changes in HDL-C and triglyceride levels. These findings mostly agree with our previous findings in an independent sample (Lee et al. 2013, 2015). We concluded then that the correlation between metabolic disturbance, clinical severity, and inflammatory aberrations might support the hypothesis that inflammation is the common underlying pathogenesis for the frequently comorbidity of BD and metabolic disturbances. In addition, because peripheral CRP levels might indicate central inflammation in the brain, Zhu et al. (2006) reported that the inflammation might decrease neurotrophic support and lead to brain dysfunction. By demonstrating concurrent increase in plasma BDNF, it is possible that the elevated plasma BDNF level observed is a compensatory response to the process of inflammation and metabolic disturbance. Our hypothesis is supported by Noren Hooten et al. (2015), who suggested that BDNF might attenuate the inflammatory stress related to high levels of CRP. The correlation between BDNF and CRP with metabolic parameters in BD requires additional investigation.

Our study has some limitations. First, we sampled only peripheral BDNF and cytokines instead of central nervous system samples because the former samples were easier to obtain. In addition, it has been suggested that changes in central BDNF and cytokine secretion might be partly reflected by the changes in peripheral levels (Pan et al. 1998; Pan and Kastin 1999). We did not explore other factors, such as smoking, diet, health supplements, or weight, all of which can affect the correlation between metabolic profiles and proinflammatory factors. Second, our study sample is small. The difference in main reduction in HDRS between the four treatment group is small. Therefore, our result should be interpreted with caution for the effect of the combination group may not have clinical superiority or significance. Studies with larger samples are needed to confirm our findings. Furthermore, it is possible that other medication permitted in the study obscured the mood stabilizing effect of the add-on medication. We did not take a ceiling effect into consideration when planning the study; however, it does appear that the patients recruited were not severely depressed. In future studies, to avoid a ceiling effect, it will be important to include patients with more severe symptoms of depression. Although we tried to limit concomitant treatment medication to only three drugs, however, we did not record previous treatment the patients were on before entering the trial; the possible anti-inflammatory effect of the add-on DM and MM interpreted from our findings should still be taken with caution. In addition, there is only very limited data supporting the effect of valproate for treatment of bipolar depression. Future study including the use of lamotrigine, a reliable treatment for bipolar depression, is needed. Because the present study was a fixed-dose comparison without dose-assessment trials, the definitive effects of add-on DM and MM and their clinical efficacy require additional studies. Finally, although we had a severity criterion, the patients recruited were not in a specific mood state, in addition, we utilized the 2-day hypomanic duration for the diagnosis of BP-II, the generalizability of our result is therefore uncertain. It will be better to refer our study group as “bipolar spectrum patients” to avoid misleading.

## Conclusion

We found that treating BD-II patients with a combination of add-on DM30 + MM5 might attenuate symptoms of depression and raise plasma BDNF levels compared with placebo, but that it had little effect on other cytokines and metabolic profiles. Add-on DM30 and MM5 alone did not have the same effect as did add-on DM30 + MM5 combined. We also found significant associations between changes in plasma BDNF and cytokine levels and changes in certain metabolic parameters, which suggested that inflammation might be the common pathogenesis between metabolic disturbance and BD. Our positive findings warrant further studies with larger samples.

## Data Availability

The datasets analyzed in the current study are available from the corresponding author on reasonable request.
